# A Systematic Review of Non-invasive Brain Stimulation Applications to Memory in Healthy Aging

**DOI:** 10.3389/fneur.2020.575075

**Published:** 2020-10-19

**Authors:** Robin A. Goldthorpe, Jessica M. Rapley, Ines R. Violante

**Affiliations:** School of Psychology, University of Surrey, Guildford, United Kingdom

**Keywords:** non-invasive brain stimulation, ageing, older adult's, memory, transcranial magnetic stimulation, transcranial direct current stimulation, transcranial alternating current stimulation, systematic review

## Abstract

It has long been acknowledged that memory changes over the course of one's life, irrespective of diseases like dementia. Approaches to mitigate these changes have however yielded mixed results. Brain stimulation has been identified as one novel approach of augmenting older adult's memory. Thus far, such approaches have however been nuanced, targeting different memory domains with different methodologies. This has produced an amalgam of research with an unclear image overall. This systematic review therefore aims to clarify this landscape, evaluating, and interpreting available research findings in a coherent manner. A systematic search of relevant literature was conducted across Medline, PsycInfo, Psycarticles and the Psychology and Behavioral Sciences Collection, which uncovered 44 studies employing non-invasive electrical brain stimulation in healthy older adults. All studies were of generally good quality spanning numerous memory domains. Within these, evidence was found for non-invasive brain stimulation augmenting working, episodic, associative, semantic, and procedural memory, with the first three domains having the greatest evidence base. Key sites for stimulation included the left dorsolateral prefrontal cortex (DLPFC), temporoparietal region, and primary motor cortex, with transcranial direct current stimulation (tDCS) holding the greatest literature base. Inconsistencies within the literature are highlighted and interpreted, however this discussion was constrained by potential confounding variables within the literature, a risk of bias, and challenges defining research aims and results. Non-invasive brain stimulation often did however have a positive and predictable impact on older adult's memory, and thus warrants further research to better understand these effects.

## Introduction

With the average life expectancy amongst most Westernised countries consistently rising (1), it has been increasingly documented that ageing is associated with certain memory changes (2). Although deterioration in memory performance can arise as a consequence of dementia, not all memory deterioration is pathological. Indeed, memory changes are widely reported amongst healthy older adults, with myriad studies identifying memory changes in individuals ageing typically with evidence of no dementia (3–5). Consequently, older adults often have a considerable degree of worry over how their memory will change as they continue ageing (6) a concern which could be valid given the above evidence of age-associated memory changes.

Research also consistently demonstrates that memory is not a unitary structure. Critical contributions to the field include: Squire et al. (7), who provided a dichotomy between episodic memories (autobiographical memories of events bound within a certain temporal context) and semantic memories (general knowledge about the world that are not time bound); Baddeley (8), who evidenced the importance of working memory (short-term storage of information for immediate information processing and manipulation); and Suzuki's (9) definition of associative memory as the ability to learn and remember relationships between unrelated items. Furthermore, such divisions of memory appear to change differentially as we age (10, 11), suggesting that ageing does not fundamentally impair memory in its entirety.

Despite this, what is clear amongst older adults is that these memory changes are noticeable and, perhaps more importantly, aversive. For example, Parikh et al. (12) found healthy older adults often identify themselves as making common memory mistakes like forgetting names or faces, which causes them to feel upset, embarrassed, and could impact upon their ability to work. Further research has relayed a similar message, with as many as half of healthy older adults worrying about their everyday memory (13). Attention has therefore turned to identifying if it is possible to ameliorate, or indeed prevent, such age-related changes in memory performance. Approaches to doing this have been varied with mixed results, ranging from talking therapy (14), pharmacological interventions (15) to the use of nutritional supplements (16). Cognitive training interventions are a further example, which have shown some positive effects when using specific tasks and when assessing memory using certain tools [e.g., (17, 18)]. Research across these fields is hence rife, with all such approaches aiming to reduce the functional impact of memory changes over the lifespan.

Amongst this corpus of research, one approach gaining momentum is non-invasive brain stimulation. Whilst brain stimulation has historically involved invasive procedures, and thus often been targeted only at clinical populations, methodological advances have dramatically reduced the invasiveness of these procedures, making such research increasingly safe (19). Given this, brain stimulation has increasingly gained popularity due to its unique ability to transiently and non-invasively modulate neuronal activity, offering the potential to safely modulate underlying neural processes potentially contributing toward memory faculties (20).

Common non-invasive brain stimulation methodologies include repeated transcranial magnetic stimulation (rTMS), transcranial direct current stimulation (tDCS), and transcranial alternating current stimulation (tACS). Whilst a full review of these is beyond the scope of this paper, a methodological review can be found within Miniussi et al. (21). Briefly, rTMS induces local neuronal depolarisation by inducing an electromagnetic field over a target area of the brain (via the scalp), which is thought to offer a high degree of temporal and spatial specificity (22). Contrarily, tDCS utilises a weak direct current over the scalp, modulating membrane potentials via voltage-gated ion channels. Such stimulation can either augment or inhibit local activity [with the association between polarity and excitation/inhibition being a matter of current debate, (23)], however typically with reduced spatial and temporal acuity to rTMS. tACS similarly induces a current via electrodes placed on the subjects' scalp however differs from tDCS by utilising an alternating current in which the electrical current periodically reverses direction. In doing so, tACS creates specific frequencies, enabling it to entrain brain oscillations and modulate associated cognitive functions (24–26). Whilst the above briefly summarises three common methodologies other variants of these also exist.

Largely prefaced in research using deep brain stimulation and increasing attempts to map cognitive faculties to regions of the brain (27), the advent of non-invasive brain stimulation has led to numerous studies exploring the role of neurostimulation in modulating memory. However, given the relative primacy of this research area, there has been little co-ordination amongst research laboratories in stimulation methodologies and memory faculties assessed. This has led to a somewhat convoluted image, with different laboratories, using different methodologies, asserting contradictory findings, and espousing contradictory conclusions [e.g., (28, 29)]. Given the potential of non-invasive brain stimulation, this is unfortunate as these inconsistencies undermine the potential to support those with memory difficulties or concerns (13). Evidently, it is therefore necessary to systematically review this growing body of research to clarify what effect such stimulation may have on older adult's memory.

Efforts have previously been made to begin systematically exploring this body of research. For example, Hsu et al. (30) performed a systematic review with both healthy older adults and patients with Alzheimer's Disease, exploring primarily the growing evidence for tDCS in modulating cognition. Authors have subsequently reviewed numerous related areas, such as the role of tDCS in improving working memory amongst the general population (31), as well as less systematic reviews of the role non-invasive brain stimulation may have in Mild Cognitive Impairment (32). Nevertheless, the literature base for non-invasive brain stimulation has grown significantly within the past 5 years, with no extant review covering the unique effects non-invasive brain stimulation has upon different aspects of healthy older adult's memory. This is surprising, given the above evidence that older adults have specific concerns about their memory, are most likely to demonstrate memory issues (33), and are thus a logical future candidate for non-invasive brain stimulation should the research allude to this.

This systematic review therefore aims to explore the current literature regarding the efficacy of non-invasive brain stimulation in augmenting healthy older adult's memory faculties. Given the (current) relative primacy of this field, this systematic review has a deliberately broad scope, considering all methodologies utilising non-invasive brain stimulation and all memory modalities. In doing so, this review hopes to gleam a greater understanding into which aspects of memory are particularly amenable to modulation from non-invasive brain stimulation, what these effects look like, and which methodologies best create and capture these effects. This would serve to create a greater unity amongst researchers in their cumulative search to greater understand the nature of memory changes during the ageing process, and potentially how to intervene.

## Methods

This systematic review has been conducted in accordance with the Preferred Reporting Items for Systematic Reviews and Meta-Analyses (PRISMA) statement (34).

### Database Searches

To identify relevant papers, Medline (1966–11 November 2019), PsycInfo (1967–11 November 2019), Psycarticles (1988–11 November 2019), and Psychology and Behavioral Sciences Collection (1965–11 November 2019) were evaluated using EBSCO host. This was set to include both published research and research pending full publication, and time frames were based upon the earliest articles for each journal available on EBSCO host to the present day. Search criteria used were [Non-invasive Brain Stimulation OR tDCS OR tACS OR TMS OR TES] AND [Older Adults OR Elderly OR Seniors OR Geriatrics] AND [Memory] to assess primary research exploring the impact of non-invasive brain stimulation on healthy older adult's memory. Search terms were set to search the entire body of academic papers for these terms to identify the greatest possible number of hits. Two of the authors of this paper first screened each paper for its relevancy by analysing titles or abstracts, which was followed by a review of each paper's full text. One additional paper was included following running this search (35), as the research laboratory were aware this had just been released, matched the search criteria, contained pertinent findings, however was not yet identifiable on EBSCO host due to its recent publication. Reinhart and Nguyen had no affiliation to the authors of this systematic review.

Studies selected for the final review were written in English in the above databases, meaning any articles not translated by said databases could not be identified by this review. To enhance the scope of this review, research was included irrespective of if they were published within a journal or thesis. Studies were included if they met the following criteria: (a) were primary research (i.e., not a review, systematic review, meta-analysis); (b) measured healthy older adults not diagnosed with dementia, psychiatric illness, ongoing physical health issue, or Mild Cognitive Impairment; (c) utilised any form of non-invasive brain stimulation: (d) actively measured memory performance, and; (e) utilised a control condition, or another mean, of assessing the unique effects of stimulation on older adults memory performance.

### Data Extraction

**Figure 1:**
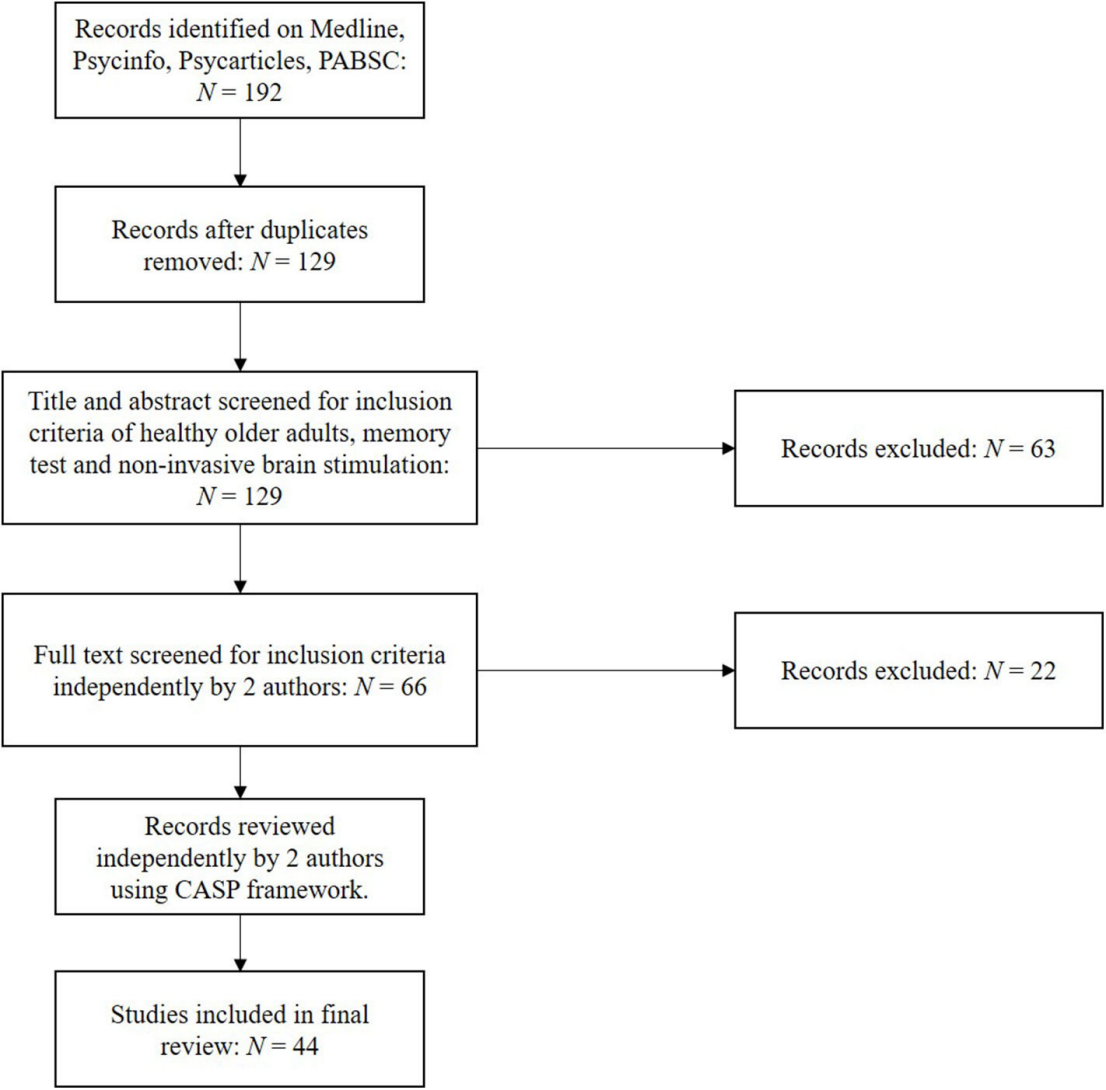
PRISMA chart for included studies.

Using the above protocol, studies were selected independently by two authors, with discrepancies submitted to the third author for review. Following extracting relevant papers, research quality was assessed independently by two authors using the Critical Appraisal Skills Programme (CASP) framework for assessing randomised controlled trials (36). This was used to address questions such as the validity and risk of bias within and across those papers included. Stimulation methodology and outcomes as assessed by at least one performance-based memory test were subsequently extracted, including significance testing and effect sizes. Forty-four original studies were identified using the above strategy, as illustrated in Figure 1.

## Results

### Study Selection and Characteristics

Using the search protocol, 192 studies were initially identified matching the search criteria, which reduced to 129 after the removal of duplicates. After screening, this was reduced to 44 papers assessed as applicable for this review's inclusion criteria. Papers were subsequently screened for their quality and risk of bias using CASP, of which each papers score (spanning 1–9, with 9 being of best quality) are presented in Tables 1–**5**. Quality of reports were all generally good, with none included scoring below 7.

**Table 1:** Working memory research.

**Author**	**Older adults sample (Age)**	**Design**	**Methodology**					**Findings**	**Significance and effect size**	**CASP quality score**
			**Stimulation**	**Control**	**Stimulation site**	**Return electrode**	**Task**			
Arciniega et al. (37)	31 (M = 67.7)	Single Blind Within Subjects	Online tDCS (2mA) 3 sessions	Sham−20 s	Right PFC (F6), or bilateral PFC (F6-F5)	Right PFC (F6)	Spatial item location	• Right tDCS (F6-P6) significantly improved task performance compared to bilateral stimulation (F6-F5)^a^. • No significant difference in recognition of visual scenes at follow up.	• ^a^*p* = 0.003, medium-large effect size	8
Berryhill and Jones (38)	25 (M = 63.7)	Within Subjects	Offline atDCS (1.5 mA) 3 sessions	Sham-20 s	PFC (F3 or F4)	Contralateral cheek	2-back	• tDCS improved working memory performance across sites in older adults in high education^a^ but not those in low education groups.	• ^a^*p* = 0.02, medium-large effect size, (ηp^2^ = 0.21)	8
Borghini et al. (39)	25 (M = 69.1)	Double Blind Within Subjects	Online tACS (1.5 mA−4 Hz, 10 Hz, 35 Hz) 4 sessions	Sham-4 Hz, 20 s	Bilateral Parietal regions	–	Object manipulation task	• Alpha-tACS significantly improved performance^a^ to a level comparable to younger adult's performance. • No significant effects of tACS on performance in theta, gamma and sham condition.	• ^a^*p* < 0.001, large effect size (*d* = 0.98)	8
Cespón et al. (40)	14 (M = 70.2)	within subjects	Offline atDCS (1.5 mA) 3 sessions	Sham-10 s	Left DLPFC (F3)	Right shoulder	n-back	• Older adults showed greater accuracy after tDCS^a^ and showed an amplified P300 event-related potential (ERP)^b^.	• ^a^*p* = 0.029 ^b^*p* = 0.021	7
Cespón et al. (41)	14 (M = 70.2)	Within Subjects	Offline tDCS (1.5 mA) 3 sessions	Sham-10 s	Left DLPFC (F3)	Right shoulder	n-back	• No significant main effect of stimulation on memory performance, however in healthy older adults after anodal tDCS, there were significant correlations between improved accuracy in n-back task and increased P300 within the left^a^ and right frontal regions^b^.	• ^a^Left (*p* = 0.05) ^b^Right (*p* = 0.04)	7
Deldar et al. (28)	15 (M = 64.0)	Double Blind Within Subjects	Online atDCS (2 mA) 2 sessions	Sham- 40 s	Left DLPFC (F3)	Right deltoid muscle	n-back	• Anodal tDCS significantly reduced reaction time in 2-back task compared to baseline.	• *p* < 0.01, medium effect size	9
Di Rosa et al. (42)	21 (M = 69.7)	Single Blind Within Subjects	Online atDCS (1.5 mA) 3 sessions	Sham-30 s	Left PFC (between F3 and F7)	Contralateral shoulder	Visuospatial Working Memory	• Compared to baseline, there was a significantly reduced reaction time in task during and after atDCS^a^. No significant differences found in sham condition. • There was increased hemodynamic activity in the bilateral PFC during and after the anodal tDCS compared to sham^b^.	• ^a^*p* < 0.05, medium effect size (ηp^2^ = 0.15) • ^b^*p* < 0.05	9
Emonson et al. (43)	19 (M = 65.5)	Between Subjects	Offline tDCS (0.7 mA) 1 session	No control	DLPFC (F7 or F8)	Contralateral supraorbital area	2-back, picture location learning and set shifting executive functioning	• No significant effect of stimulation between age groups after each task. • Improvement across all task conditions (not significance tested).	• N. S.	7
Jones et al. (44)	72 (M = 64.4)	Single BlindBetween Subjects	Offline atDCS (1.5 mA) 10 sessions	Sham-20 s	PFC (F4), PPC (P4), or alternation anodal PFC and PPC.	Contralateral cheek	Digit Span, Stroop and spatial 2-back	• All groups benefited from 10 working memory training sessions. • After 1 month the tDCS group showed significantly better performance in tasks compared to sham^a^. • No significant differences between different sites stimulated.	• ^a^*p* < 0.01, medium effect size (ηp^2^ = 0.10)	9
Nilsson et al. (45)	30 (M = 69.0)	Single Blind Within Subjects	Offline tDCS (1 or 2 mA) 3 sessions	Sham-40 s	Left DLPFC (F3)	Contralateral supraorbital area	n-back	• No significant effect of stimulation across conditions and time points.	• N. S.	8
Nilsson et al. (29)	123 (M = 69.7)	Double BlindBetween Subjects	Offline atDCS (2 mA) 19 sessions	Sham-30 s	Left DLPFC (F3)	Contralateral supraorbital area	Updating and set- switching	• No significant effects of tDCS on memory performance over sham.	• N. S.	9
Park et al. (46)	40 (M = 69.7)	Double BlindBetween Subjects	Online atDCS (2 mA) 10 sessions	Sham-30 s	Bilateral PFC (F3 and F4)	Non-dominant arm	Verbal Working Memory	• Compared to baseline, there was a significant increase of task accuracy and faster reaction time in tDCS group immediately following training (T1)^a^. No significant differences in sham condition. • Effects maintained after 4 weeks (T2) compared to baseline for the tDCS group, showing significantly increased accuracy and quicker reaction time^b^.	• ^a^Accuracy T1 (*p* = 0.04); RT T1 (*p* = 0.05) • ^b^Accuracy T2 (*p* = 0.002); RT T2 (*p* = 0.018)	9
Reinhart and Nguyen (35)	42 (M = 68.8)	Double Blind Within Subjects	Online tACS (Tuned to pp. unique theta frequency, or 8 Hz non-tuned) 3 sessions	Sham-30 s	Left PFC and left temporal cortex simultaneously		Visual Working Memory	• Compared to sham, theta stimulation significantly improved working memory performance in older adults, with effects lasting up to 50 min post-stimulation^a^. This co-occurred with increased phase-amplitude coupling in frontotemporal regions (measured via EEG). • Frontal or temporal theta stimulation alone did not significantly augment working memory, neither did 8 Hz non-tuned stimulation.	• ^a^*p* = 0.001, medium effect size (*d* = 0.57)	8
Stephens (47)	90 (M = 69.0)	Single BlindBetween Subjects	Offline atDCS (1 or 2 mA) 7 sessions	Sham - 20 s	Right PFC (F4)	Contralateral cheek	Far transfer tasks: WAIS coding, go/no-go, functional maths problems	• After 1 month and working memory training, those who received 2 mA tDCS performed significantly better on far transfer tasks compared to those who revieved 1 mA tDCS and sham^a^. No significant differences between 1 mA tDCS and sham groups.	• ^a^*p* = 0.018, medium-large effect size	9
Stephens et al. (48)	137 (M = 66.7)	Single BlindBetween Subjects	Offline atDCS (1, 1.5 or 2 mA) 10 sessions	Sham	PFC (F4) or PPC (P4) or alternationof PFC and PPC.	Contralateral cheek	Spatial working memory and picture recognition	• After one session of tDCS, significant group differences in task performance were predicted by COMT val158met status^a^. • After 1 month, there was a significant interaction of tDCS intensity, COMT genotype, and task^b^. This showed dose dependent tDCS effects with 1-1.5mA tDCS having the greatest gains in spatial performance.	• ^a^*p* = 0.002, medium effect size (ηp^2^ = 0.10) • ^b^*p* = 0.03, medium-effect size (ηp^2^ = 0.10)	8
Stoynova et al. (49)	26 (M = 68.9)	Single Blind,Between Subjects	Online tDCS (2 mA) 14 sessions	Sham	Left DLPFC (F3)	Right deltoid muscle	Auditory addition	• tDCS with cognitive training significantly reduced memory concerns compared to sham immediately after training.	• *p* = 0.018, large effect (*d* = 1.0)	8
Yamanaka et al. (50)	38 (M = 72.4)	Single Blind Within Subjects	Online TMS (50 μV−5 Hz) 4 sessions	No Stimulation	Left PPC (P3), or Right PPC (P4)		Spatial working memory	• In older adults, mean RT was significantly shorter when stimulating P3 vs. P4^a^, however overall stimulation did not significantly improve older adult's performance.	• ^a^*p* = 0.01	8

*CASP scores range from 0 to 9, with 0 being lowest possible quality and 9 being highest possible quality. atDCS, anodal tDCS; DLPFC, dorsolateral prefrontal cortex; N.S., not significant; PFC, prefrontal cortex; PPC, posterior parietal cortex; RT, reaction time*.

All included papers recruited older adult populations, which were assessed as healthy using multiple approaches. For clarity, results have been divided into the memory domain assessed. This was due to the high degree of overlap between certain study's methodology/outcomes, with methodologies used clustered together within each sub-section. Online stimulation refers to stimulation delivered during the task(s) of interest, while offline stimulation refers to delivery outside the task(s) of interest, generally before the task and at rest.

### Working Memory

Working memory, as defined as the ability to store, process, and manipulate short-term information was assessed within 17 studies. These are summarised in Table 1. By far the most common stimulation method was tDCS (14 papers), of which 10 studies found significant benefits on working memory performance (28, 37, 38, 40, 42, 44, 46–49), and 4 did not (29, 41, 43, 45). Across all studies the region most commonly stimulated was the prefrontal cortex (PFC).

Deldar et al. (28) and Stoynova et al. (49) both reported positive findings when targeting the left dorsolateral prefrontal cortex (DLPFC) with online tDCS during working memory tasks; Stoynova et al. evidenced enhanced self-confidence on an auditory addition task across 12 stimulation sessions, and Deldar et al. evidenced a decreased mean reaction time on a modified N-Back task over 1 stimulation session compared to sham. Cespón et al. (40) also identified greater accuracy on the N-Back task, however DLPFC stimulation here was done immediately before the task.

Di Rosa et al. (42) found faster reaction times during and after stimulation of the left PFC on a visuo-spatial working memory paradigm, which corresponded with increased hemodynamic activity bilaterally in the PFC. Arciniega et al. (37) found that right PFC-posterior parietal (PPC) online stimulation (as compared to sham or bilateral PFC stimulation) improved visual working memory in an item location paradigm. Stephens (47) stimulated the right PFC over 5 cognitive training sessions, and found that tDCS uniquely improved performance on novel working memory tasks up to a month afterwards. Finally, Park et al. (46) identified that bilateral PFC stimulation can benefit verbal working memory, with tDCS significantly augmenting cognitive training for up to 4 weeks on verbal working memory paradigms.

Other studies have evidenced the effects of tDCS to be less tied to region-specific stimulation. Stephens et al. (48) identified that just one session of tDCS across different sites augmented working memory performance. Moreover, this research went further in identifying a particular variant of the COMT gene predicted tDCS efficacy, with effects lasting up to 1 month post-stimulation and training. Jones et al. (44) similarly found offline non-site-specific stimulation was sufficient to improve visuo-spatial working memory, with improved performance both after 10 sessions of training and after a 1 month delay. A final nuanced effect was observed by Berryhill and Jones (38), as although these authors stimulated frontal regions offline, significant improvements on the N-back task were only observed amongst participants identified as being more highly educated.

Four papers failed to identify a significant effect of tDCS on working memory performance. Cespón et al. (41) failed to identify a significant change in reaction time on the N-back task amongst healthy older adults immediately following DLPFC stimulation, and found no unique memory benefits following stimulation [a result that appears to oppose their previous findings; (40)]. That said, anodal tDCS did result in increased P300 amplitudes (a marker of cognitive processing measured using electroencephalography), which correlated with improved accuracy. Emonson et al. (43) failed to identify a significant improvement on the N-Back task following one session of DLPFC stimulation, however used a weak current of 0.7 mA (lower than the above studies). In two studies, Nilsson et al. (29, 45) failed to find an improvement in working memory performance during or after up to 20 rounds of tDCS stimulation of the left DLPFC.

Three studies used other forms of brain stimulation. Yamanaka et al. (50) used rTMS to assess working memory via a delayed match-to-sample task, with stimulation of either the left or right parietal regions [P3 and P4, respectively, based on the electroencephalography (EEG) International system; (78)]. No significant improvements were found when stimulation was delivered in 5 Hz pulses during the task, however P3 stimulation did significantly reduce reaction times relative to P4.

Reinhart and Nguyen (35) utilised online individually-tuned theta-frequency tACS in a phase-synchronous manner over temporal and frontal regions. In doing so, this study identified significant improvements in older adults visual working memory both during and for up to 50 min post-stimulation. In addition, EEG recordings showed increased post-stimulation phase-amplitude coupling in frontotemporal regions, however only when stimulation was phase-synchronised across fronto-temporal sites and performed at theta frequency. Finally, Borghini et al. (39) showed that online alpha-tACS uniquely augmented performance on a visual working memory paradigm (compared to theta or gamma frequency), increasing performance to a level comparable with healthy young adults.

### Associative Memory

Associative memory, as defined as the ability to learn and remember the relationship between previously unrelated items, was assessed within 14 original papers (presented in Table 2). Anodal tDCS was the most common mode of modulating brain activity, with 8 papers employing this approach. Of these, 4 identified a statistically significant result (51, 52, 55, 58), whilst 4 did not observe a main effect of tDCS on memory performance (43, 56, 59, 62).

**Table 2:** Associative memory research.

**Author**	**Older adults sample (Age)**	**Design**	**Methodology**					**Findings**	**Significance and effect size**	**CASP quality score**
			**Stimulation**	**Control**	**Stimulation site**	**Return electrode**	**Task**			
Antonenko et al. (51)	20 (M = 70.0)	Single BlindBetween Subjects	Online atDCS (1 mA) 3 sessions	Sham-30 s	Right Temporoparietal Area (T6)	Contralateral supraorbital cortex	Object location learning	• Younger adults outperformed older adults across all conditions^a^. • Overall, atDCS condition had better task peformance compared to sham^b^.	• ^a^*p* = 0.002 • ^b^*p* = 0.014	8
Antonenko et al. (52)	34 (M = 63.1)	Within Subjects	Online atDCS (1 mA) 3 sessions	Sham−30 s	Left temporoparietal cortex	Right supraorbital area	Pseudo word-object pair task	• Improved immediate and delayed (20 min.) recall of associations in tDCS group compared to sham^a^. • Steeper learning curves in tDCS group compared to sham^b^.	• ^a^*p* = 0.014 • ^b^*p* = 0.014	8
Davis et al. (53)	15 (M = 67.2)	Within Subjects	Offline rTMS (1 or 5 Hz) 2 sessions	No control	Left Middle Frontal Gyrus		Word-pair task	• No significant differences between stimulation groups in both memory performance and reaction time to correct trials.	• N. S.	8
Eggert et al. (54)	26 (M = 69.1)	Double Blind Within Subjects	Offline sotDCS (260 μA, 0.75 Hz) 2 sessions	No Stimulation	Bilateral PFC (F3 and F4)	Ipsilateral mastoids	Word-pair association	• No significant effects of stimulation on performance in memory tasks. Performance deteriorated similarly across groups.	• N. S.	8
Emonson et al. (43)	19 (M = 65.5)	Between Subjects	Offline tDCS (0.7 mA) 1 session	No control	DLPFC (F7 and F8)	Contralateral supraorbital area	Picture location learning	• No significant effect of stimulation between age groups after each task. • Improvement across all tasks (not significance tested).	• N. S.	7
Flöel et al. (55)	20 (M = 62.1)	Double Blind Within Subjects	Online atDCS (1 mA) 2 sessions	Sham-30 s	Right Temporoparietal Area (T6)	Contralateral supraorbital area	Object location learning	• No significant differences in performance between conditions immediately. • After 1 week, free recall significantly improved in tDCS condition compared to sham^a^.	• ^a^*p* < 0.05	9
Külzow et al. (56)	32 (M = 68.0)	Single blind Within subjects	Online atDCS (1 mA) 3 sessions	Sham-30 s	Right Temporoparietal Area (T6)	Contralateral supraorbital area	Object location learning	• Training success and delayed memory was not affected by atDCS. • On day 3, visuospatial training significantly improved task performance independent of atDCS^a^.	• ^a^Large effect size (*d* = 0.70)	9
Ladenbauer et al. (57)	18 (M = 65.0)	Single blind Within subjects	Offline sotDCS (260 μA, 0.75 Hz) 3 sessions	No Stimulation	Bilateral PFC (F3 and F4)	Ipsilateral mastoids	Object location learning, word-pairs	• sotDCS significantly increased frontal slow oscillatory activity^a^ and fast spindle activity^b^ compared to sham.	• ^a^*p* = 0.029 • ^b^*p* = 0.003	9
Leach et al. (58)	14 (M = 71.7)	Double blindBetween subjects	Online atDCS (2 mA) 1 session	Sham- 0.1 mA	Left inferior PFC (F9)	Contralateral upper arm	Face-name	• False alarm rates were significantly higher for tDCS condition compared to sham^a^, therefore performance was decreased with use of tDCS. • No significant differences between stimulation groups for free recall.	• ^a^*p* < 0.05	9
Leach et al. (59)	48 (M = 65.6)	Double blindBetween subjects	Online atDCS (1.5 mA) 2 sessions	Sham- 0.1 mA	Left DLPFC (F3)	Contralateral upper arm	Face-name	• No significant effect of stimulation on recall and recognition performance in older adults. Only significant effects found in younger adults.	• N. S.	8
Manenti et al. (60)	31 (M = 68.6)	Within subjects	Online rTMS (20 Hz) 1 session	Sham	Left or right DLPFC (BA 46)		Word-pair task	• Interference caused by left DLPFC stimulation was significantly higher when applied during encoding compared to retrieval^a^. • During encoding, there were significant differences in rTMS effects^b^, showing a predominance of the left DLPFC present in a LP (low performance) group. • No significant differences for HP (high performance) group, showing right rTMS effects for both encoding and retrieval.	• ^a^*p* = 0.047 • ^b^*p* = 0.001	7
Paßmann et al. (61)	21 (M = 65.0)	Single blind Within subjects	Offline sotDCS (260 μA, 0.75 Hz) 2 sessions	No Stimulation	Bilateral PFC (F3 and F4)	Ipsilateral mastoids	Object location learning, Word-pair task	• Increased slow oscillatory activity, after sotDCS compared to sham stimulation, for both prefrontal^a^ and frontal electrode^b^ sites. • Increase in power in the spindle frequency bands after so-tDCS compared to sham, for both frontal and prefrontal regions.^c^ • No significant change in object location learning or word-pair learning.	• ^a^*p* = 0.001 • ^b^*p* = 0.013 • ^c^prefrontal: *p* = 0.001; frontal: *p* = 0.002	9
Prehn et al. (62)	20 (M = 66.0)	Double Blind Within subjects	Online atDCS (1 mA) 4 sessions	Sham	Right Temporoparietal Area (T6)	Contralateral frontopolar cortex	Object location learning	• In both younger and older adults, performance improved by SSRI and atDCS compared to sham and placebo^a^. No significant effects of tDCS alone. • Older adults performed worse in task compared to young adults^b^.	• ^a^*p* = 0.005, medium effect size (Hedges' g = 0.45) • ^b^*p* = 0.001	9
Westerberg et al. (63)	19 (M = 73.4)	Double Blind Within subjects	Offline sotDCS (260 μA, 0.75 Hz) 2 sessions	No Stimulation	DLPFC (F7 and F8)	Ipsilateral mastoids	Word-pair recall	• Across both sessions, post-nap recall was significantly improved compared to pre-nap recall^a^. • Word-pair recall improvement, comparing pre-nap to post-nap and was larger in the sotDCS session compared to sham^b^.	• ^a^*p* < 0.01 • ^b^*p* < 0.05	8

*CASP scores range from 0 to 9, with 0 being lowest possible quality and 9 being highest possible quality. atDCS, anodal tDCS; DLPFC, dorsolateral prefrontal cortex; PFC, prefrontal cortex; N.S., not significant; sotDCS, slow oscillation tDCS*.

Amongst the 4 tDCS studies that identified statistically significant results, both Antonenko et al. (51) and Flöel et al. (55) utilised comparable stimulation protocols over the right temporoparietal region during 2–3 learning sessions, and both reported improved object-location pairing learning. However, Antonenko et al. (51) identified incremental increases in performance over a 3-day period of object-location learning, whilst Flöel et al. (55) found improved free recall of object-location pairings 1-week post-learning.

Antonenko et al. (52) showed that stimulation of the left temporoparietal cortex during learning improved both immediate and delayed recall of object-pseudo-word associations, persisting for up to 20 min post-stimulation. Notably, participants who received tDCS also showed a faster learning curve of object-pseudo-word pairings, and evidenced augmented hippocampo-temporoparietal functional connectivity, suggesting network-level effects of stimulation.

Finally, Leach et al. (58) hypothesised that older adults may as a group have diminished face-name learning, and be specifically impaired by left inferior PFC tDCS. Their findings appeared to corroborate this theory, with one session of online tDCS causing a reduction in accuracy during recognition trials.

Four tDCS studies did not observe significant effects. Leach et al. (59) used a face-name association paradigm and found that stimulation of the left DLPFC during the task improved recognition and recall in younger but not in older adults. Emonson et al. (43) utilised a weak current over the left DLPFC, and was unable to identify any significant changes in older adult's subsequent performance on an object-location association paradigm. Külzow et al. (56) used a similar stimulation paradigm, targeting instead the right temporoparietal region, and found that tDCS-paired object-location learning did initially increase after the first day of training, however this did not persist after 3 trials and overall performance did not significantly differ from sham stimulation. Prehn et al. (62) also found no significant effect of tDCS over the right temporoparietal area on an object-location association task. Prehn et al. did however find a significant effect when utilising both Selective Serotonin Reuptake Inhibitors (SSRI's) and tDCS concurrently on this task, however this was only achieved via pooling older and younger adults together.

An additional 4 studies utilised an alternative form of tDCS known as slow oscillatory tDCS (sotDCS), assessing the effects of stimulation during slow-wave sleep on associative memory performance. Of these studies, Westerberg et al. (63) used sotDCS during a nap after learning word pairings, and stimulated participants at a frequency of 0.75 Hz in 5 blocks of 5 min after the onset of stage 2 sleep. Such stimulation was targeted toward bilateral frontal lobe regions at locations F7 and F8 according to the international 10–20 EEG system (78). Whilst word recall increased across both sham and sotDCS following a nap, the increase was significantly greater amongst those who received sotDCS.

Three sotDCS studies however reported no significant improvement in associative memory (54, 57, 61). Whilst these used similar memory tasks to Westerberg et al. (63), they differed in stimulation location by applying stimulation bilaterally to the frontal lobes but with electrodes placed in areas F3 and F4 (international 10–20 EEG system). Eggert et al. (54) found that sotDCS during early non-REM sleep did not improve word-pair memory consolidation, and that across conditions memory generally deteriorated following sleep. Ladenbauer et al. (57) and Paßmann et al. (61) both found that participants recalling lists of word-pairs or object-location pairings generally performed worse following a nap, with no significant differences between sham and sotDCS.

A final 2 studies examined the effects of rTMS on associative memory performance (53, 60). Manenti et al. (60) applied rTMS (20 Hz) to either the left or right DLPFC during encoding or recalling of word-pairs and found that stimulation significantly impeded word-pair recall accuracy. By subsequently breaking down test performance into high performers and low performers, the authors also reported that online stimulation of either the left or right DLPFC impeded performance in high performers, whilst lower performers were significantly more affected by stimulation to the left than right DLPFC.

Davis et al. (53) used rTMS at lower frequencies of either 1 or 5 Hz in the left DLPFC and did not identify any subsequent changes in word-pair association memory. Using functional Magnetic Resonance Imaging (fMRI), Davis et al. did however find changes in neural activity following differing stimulation conditions. 1 Hz stimulation decreased local success-related activity, causing distributed bilateral PFC activity instead, whereas 5 Hz stimulation increased success-related local activity, resulting in increased local connectivity within the PFC.

### Episodic Memory

Episodic memory, defined as the ability to recall time-bound, personally relevant experiences, was assessed within 11 research papers (Table 3). The most commonly cited form of non-invasive brain stimulation was anodal tDCS (9 papers), of which 7 cited improved episodic memory recall (64–67, 69–71), and 2 did not (37, 68).

**Table 3:** Episodic memory research.

**Author**	**Older adults sample (Age)**	**Design**	**Methodology**					**Findings**	**Significance and effect size**	**CASP quality score**
			**Stimulation**	**Control**	**Stimulation site**	**Return electrode**	**Task**			
Arciniega et al. (37)	31 (M = 67.7)	Single Blind Within Subjects	Online tDCS (2 mA) 3 sessions	Sham-20 s	Right PFC (F6), or bilateral (F6)	Right PFC (F6)	Spatial item location	• No significant difference in recognition of visual scenes at follow up.	• N. S.	8
Brambilla et al. (64)	32 (M = 67.9)	Single blind Within Subjects	Online tDCS (1.5 mA) 2 sessions	Sham	Bilateral parietal cortex (PARC) or DLPFC	Contralateral supraorbital area	Word recognition	• Left hemisphere tDCS significantly improved performance in older adults compared to sham^a^. • When using tDCS, low performing older adults obtained significantly lower scores than young adults^b^ and high performing elderly group^c^. • When using tDCS, young adults achieved similar accuracy in word recognition to high performing older adults^d^, but were more accurate as compared to low performing older adults^e^. • Following stimulation, young adults obtained similar scores to high performing older adults.	• ^a^*p < 0.001* • ^b^*p* < 0.001; ^c^*p* < 0.001 • ^d^*p* > 0.05; ^e^*p* < 0.001	9
Ladenbauer et al. (57)	18 (M = 65.0)	Single blind Within subjects	Offline sotDCS (260 μA, 0.75 Hz) 3 sessions	No Stimulation	Bilateral PFC (F3 and F4)	Ipsilateral mastoids	Visual scene recognition	• Picture memory retention scores were improved with sotDCS after the nap compared to sham stimulation during the nap^a^. • Significantly increased frontal slow oscillatory activity^b^ and fast spindle activity^c^ in sotDCS condition compared to sham.	• ^a^*p* = 0.013 • ^b^*p* = 0.029; ^c^*p* = 0.003	9
Manenti et al. (65)	32 (M = 67.9)	Single blind Within subjects	Online tDCS (1.5 mA) 1 session	Sham	PARC or DLPFC	Contralateral supraorbital area	Word recognition	• Significantly better task performance after left tDCS application in older adults compared to both sham^a^ and to right tDCS^b^.	• ^a^*p* < 0.001 • ^b^*p* = 0.003	7
Manenti et al. (66)	22* (M = 74.5)	Double blind Between subjects	Offline tDCS (1.5 mA) 1 session	Sham- 10 s	Left lateral PFC	Right supraorbital area	Word learning	• Anodal tDCS improved accuracy of recognition of previously seen words when assessed 30 days post-learning^a^. • During free recall, no significant differences in the numbers of words correctly recalled between the anodal and sham group.	• ^a^*p* < 0.004, large effect size (*d* = 1.49)	8
Medvedeva et al. (67)	22 (M = 73.0)	Single blind Within subjects	Online and offline tDCS (2 mA) 2 sessions	Sham−30 s	left VLPFC (F7)	Contralateral deltoid muscle	Word recall	• tDCS during encoding had significantly better accuracy after 24 h compared to sham.	• *p* = 0.033, large effect size (*d* = 1.01)	9
Paßmann et al. (61)	21 (M = 65.0)	Single blind Within subjects	Offline sotDCS (260 μA, 0.75 Hz) 2 sessions	No Stimulation	Bilateral PFC (F3 and F4)	Ipsilateral mastoids	Free recall task	• Increased slow oscillatory activity, after sotDCS compared to sham stimulation, for both prefrontal^a^ and frontal electrode^b^ sites. • Increase in power in the spindle frequency bands after sotDCS compared to sham, for both frontal and prefrontal regions^c^. • Significantly impaired free recall of visual memory overnight performance after a night with so-tDCS compared to sham^d^.	• ^a^*p* = 0.001; ^b^*p* = 0.013 • ^c^prefrontal: *p* = 0.001; frontal: *p* = 0.002 • ^d^*p* = 0.036, large effect size (ηp^2^ = 0.20)	9
Peter et al. (68)	51 (M = 68.8)	Double blind Between subjects	Offline atDCS (1 mA) 1 session	Sham	DLPFC (F3)	Contralateral supraorbital area	Verbal episodic recall	• No statistical differences between active and control for older and younger adults. • Verbal delayed recall performance in younger adults significantly mediated by a reduction in negative affect following stimulation, however no such effect identified in older adults.	• N. S.	9
Sandrini et al. (69)	36 (M = 67.2)	Double Blind Between Subjects	Offline atDCS (1.5 mA) 1 session	Sham- 10 seconds	Left DLPFC (F3)	Right supraorbital area	Word learning and recall	• atDCS both with and without a reminder (R and NR) significantly improved task performance compared to sham^a^. • Significant memory decay at Day 30 in Sham-R compared to Anodal-R^b^ and Anodal-NR^c^.	• ^a^*p* = 0.02, large effect size (ηp^2^ = 0.22) • ^b^R (*p* = 0.04); ^c^NR (p < 0.01)	9
Sandrini et al. (70)	28 (M = 68.9)	Double blind Between subjects	Offline atDCS (1.5 mA) 1 session	Sham- 10 s	Left DLPFC (F3)	Right supraorbital area	Word recall	• 48 h after stimulation, the atDCS group recalled significantly more words correctly compared to the sham group^a^. • No significant group effects immediately or after 1 month.	• ^a^*p* = 0.007, large effect size (*d* = 1.01)	9
Sandrini et al. (71)	28 (M = 67.9)	Double blind Between subjects	Offline tDCS (1.5 mA) 1 session	Sham	Left DLPFC (F3)	Right supraorbital area	Word learning	• No significant differences in performance between sham and active group after 2 days. • tDCS group perform significantly better after 30 days compared to sham^a^.	• ^a^*p* = 0.026	9

*CASP scores range from 0 to 9, with 0 being lowest possible quality and 9 being highest possible quality. atDCS, anodal tDCS; DLPFC, dorsolateral prefrontal cortex; PFC, prefrontal cortex; N.S., not significant; sotDCS, slow oscillation tDCS*.

The most common means of assessing episodic memory was via word learning and recall tasks. Manenti et al. (65) identified that tDCS over the left DLPFC and parietal cortex during retrieval augmented recognition of words 5 min after being ambiguously presented with a list of words. Notably, bilateral stimulation improved recognition in younger subjects, whereas only left hemisphere stimulation augmented older adult performance. Brambilla et al. (64), utilising the same sample as Manenti et al. (65), extended this by using fMRI data to identify that high performing older adults on the task utilised bilateral parietal cortex/ DLPFC during retrieval, whilst low performers asymmetrically utilised the left hemisphere.

In a series of experiments, Sandrini et al. (69) utilised a word learning task which, after 24 h, was followed by participants seeing contextual reminders and subsequently receiving tDCS to the left DLPFC. This paradigm resulted in improved word recall after a delay of both 3 and 30 days. This finding was further corroborated by Manenti et al. (66), which used a similar paradigm and identified significant improvements in word recognition after a 30 day interval. Sandrini et al. (70) stimulated the left DLPFC whilst participants initially learned a series of words, and found that relative to sham tDCS improved recall accuracy 3 days post-learning, however not after 30 days. Sandrini et al. (71) tweaked this methodology, shifting left DLPFC tDCS to immediately after word encoding for 15 min. This resulted in no significant differences between sham and active stimulation 3 days post-learning, however significantly better performance after 30 days post-stimulation.

Medvedeva et al. (67) stimulated instead the left Ventrolateral PFC (VLPFC) during word encoding, hypothesising that this is similarly utilised during word encoding. In line with their hypothesis, Medvedeva et al. found significantly enhanced word learning 24 h post stimulation when comparing tDCS to sham.

Two tDCS protocols were not able to identify a significant effect of tDCS on episodic memory. Arciniega et al. (37) exposed participants to a series of visual scenes whilst having either bilateral or right PFC stimulation, and found no significant benefits of stimulation on recognition of familiar scenes after a short delay. Peter et al. (68) stimulated the left DLPFC for 20 min whilst learning a series of words, however found 20 min post-learning there was no significant differences between sham and DLPFC stimulation in recall amongst older adults.

Two further paradigms explored the effects of non-invasive brain stimulation on episodic memory using sotDCS (57, 61), of which both found significant effects. Ladenbauer et al. (57), during the previously cited visuo-spatial associative memory task, found that bilateral F3 and F4 stimulation during a nap significantly improved recognition memory of visual scenes presented prior to falling asleep (as compared to no stimulation). This was hypothesised to relate to boosted slow oscillatory activity during early sleep. Conversely, Paßmann et al. (61), using a similar methodology, identified that sotDCS during early sleep impaired consolidation of visual memories, significantly reducing participants accuracy when recognising visual scenes presented prior to sleep.

### Semantic Memory

Semantic memory, defined as recollection of facts and general knowledge about the world, was assessed within 3 original pieces of research. Of these papers, all used tDCS, and all 3 found significant effects of tDCS augmenting semantic memory recollection (72–74). These papers are summarised in Table 4.

**Table 4:** Semantic memory research.

**Author**	**Older adults sample (Age)**	**Design**	**Methodology**					**Findings**	**Significance and effect size**	**CASP quality score**
			**Stimulation**	**Control**	**Stimulation site**	**Return electrode**	**Task**			
Martin et al. (72)	18 (M = 68.4)	Within subjects	Online tDCS (1 mA) 3 sessions	Sham- 30 s	Right supraorbital region and right M1	Right supraorbital area or Right M1	Semantic word generation	• Overall for older and younger adults, both atDCS over the right supraorbital region^a^ and dual tDCS over right M1^b^ significantly reduced the number of errors on the semantic word retrieval task compared to sham.	• ^a^*p* < 001, large effect size (ηp^2^ = 0.28) • ^b^*p* < 0.001, large effect size (ηp^2^ = 0.26)	7
Meinzer et al. (73)	18 (M = 68.4)	Single blind Within subjects	Online atDCS (1 mA) 3 sessions	Sham−30 s	Left or bilateral M1 (C3)	Right M1 or right supraorbital area	Semantic word generation	• Significantly less errors during task in atDCS condition compared to sham when stimulating both uni-^a^ and bi-lateral M1^b^.	• ^a^*p* = 0.004, large effect size (*d* = 0.80) • ^b^*p* = 0.002, large effect size (*d* = 0.85)	9
Ross et al. (74)	14 (M = 65.0)	Within subjects	Offline tDCS (1.5 mA) 3 sessions	Sham - 30 s	Right or left Anterior Temporal Lobe (ATL, T3 and T4)	Contralateral cheek	Face naming and Location naming	• Older adults showed significant task improvement remembering famous faces after left ATL stimulation compared to sham^a^. Younger adults showed significant task improvement in face naming after right ATL stimulation compared to sham^b^. • Older adults significantly improved in location naming task in right ATL stimulation compared to sham^c^. No significant differences in left ATL stimulation compared to sham.	• ^a^*p* = 0.007, large effect size (ηp^2^ = 0.44) • ^b^*p* = 0.007, large effect size (ηp^2^ = 0.42) • ^c^*p* = 0.04	8

*CASP scores range from 0 to 9, with 0 being lowest possible quality and 9 being highest possible quality. atDCS, anodal tDCS; ATL, Anterior Temporal Lobe*.

Ross et al. (74) asked participants to name age-matched famous people or landmarks whilst concurrently having tDCS to either the left or right anterior temporal lobe (vs. sham). Whilst this research found that tDCS alone did not significantly improve recognition, on trials where participants took longer to respond, stimulation to the left anterior temporal lobe significantly improved face recognition, whilst stimulation to the right anterior lobe significantly improved landmark recognition.

Meinzer et al. (73) assessed the effects of either left or bilateral tDCS to the primary motor cortex during a semantic word generation task. Both stimulation paradigms significantly improved accuracy vs. sham, with neither significantly outperforming the other. Martin et al. (72) also assessed the effects of tDCS to the primary motor cortex during a semantic word generation task. They found that whilst performance significantly improved for both younger and older adults, for older adults this increment was more pronounced. Moreover, in older adults such stimulation uniquely caused greater left laterality in processing, modulating network-level neural dynamics.

### Procedural Memory

Procedural memory, which is the ability to learn and remember motor skills, typically outside of conscious awareness, was assessed within 6 papers (summarised in Table 5). Of these papers, 2 utilised anodal tDCS, and both identified significant effects of tDCS in augmenting procedural memory (75, 76).

**Table 5:** Procedural memory research.

**Author**	**Older adults sample (Age)**	**Design**	**Methodology**					**Findings**	**Significance and effect size**	**CASP quality score**
			**Stimulation**	**Control**	**Stimulation site**	**Return electrode**	**Task**			
Eggert et al. (54)	26 (M = 69.1)	Double blind Within subjects	Offline sotDCS (260 μA, 0.75 Hz) 2 sessions	No Stimulation	Bilateral PFC (F3 and F4)	Ipsilateral mastoids	Procedural memory	• No significant effects of stimulation on performance in memory tasks. Performance deteriorated similarly across groups.	• N. S.	8
Ladenbauer et al. (57)	18 (M = 65.0)	Single blind Within subjects	Offline sotDCS (260 μA, 0.75 Hz) 3 sessions	No Stimulation	Bilateral PFC (F3 and F4)	Ipsilateral mastoids	Motor sequence task	• Significant increase in frontal slow oscillatory activity^a^ and fast spindle activity^b^ in sotDCS condition compared to sham. • No other significant results.	• ^a^*p* = 0.029 • ^b^*p* = 0.003	9
Parikh (75)	8 (M = 75.0)	Single blind Within subjects	Online atDCS (1 mA) 2 sessions (1 anodal, 1 sham)	Sham	Left M1	Right supraorbital area	Pegboard fine motor control	• 35 min after stimuation, the atDCS group significantly improved on the task, whilst sham significantly deteriorated back toward baseline performance.	• *p* < 0.025	7
Paßmann et al. (61)	21 (M = 65.0)	Single blind Within subjects	Offline sotDCS (260 μA, 0.75 Hz) 2 sessions	No Stimulation	Bilateral PFC (F3 and F4)	Ipsilateral mastoids	Motor sequence task	• Increased slow oscillatory activity, after sotDCS compared to sham stimulation, for both prefrontal^a^ and frontal electrode^b^ sites. • Increase in power in the spindle frequency bands after sotDCS compared to sham, for both frontal and prefrontal regions.^c^ • No significant change in motor sequencing.	• ^a^*p* = 0.001; ^b^*p* = 0.013 • ^c^prefrontal: *p* = 0.001; frontal: *p* = 0.002	9
Rumpf et al. (76)	100 (M = 65.4)	Double blind Within subjects	Offline tDCS (1 mA) 3 sessions	Sham - 30 s	Left M1 (C3) or premotor cortex	Supraorbital area ipsilateral to the trained hand	Motor sequence learning	• Performance was modulated by the type of post-training tDCS^a^. • Anodal tDCS on M1 significantly improved immediate performance after 8 h and 1 day compared to cathodal M1 stimulation^b^, anodal PMC stimulation^c^ and sham^d^. If stimulation delayed by 60 or 120 min, this effect does not occur.	• ^a^*p* = 0.004, large effect size (*d* = 1.20) • ^b^aM1 vs. cM1 (*p* = 0.010) • ^c^aM1 vs. aPMC (*p* = 0.001) • ^d^aM1 vs. sham (*p* = 0.004)	8
Rumpf et al. (77)	33 (M = 67.7)	Double blind Within subjects	Offline tACS (1 mA−10 or 20 Hz) 2 sessions	Sham	Left M1 (C3)	Right supraorbital area ipsilateral to trained hand	Motor sequence learning	• 6 h after training, performance was significantly impaired in alpha-tACS condition compared to sham^a^. • No significant change in consolidation in beta-tACS compared to sham.	• ^a^*p* = 0.037	9

*CASP scores range from 0 to 9, with 0 being lowest possible quality and 9 being highest possible quality. atDCS, anodal tDCS; DLPFC, dorsolateral prefrontal cortex; M1, primary motor cortex; N.S., not significant; sotDCS, slow oscillation tDCS*.

Parikh (75) stimulated the primary motor cortex approximately over the hand region (M1) as participants practiced completing a pegboard motor task. Whilst practice improved performance independent of stimulation condition, only those who received M1 tDCS maintained these gains after a delay of 35 min across numerous measures of fine motor control on this task.

Rumpf et al. (76) stimulated the left primary or premotor cortex (vs. sham) either immediately or after a short period following completing a finger tapping sequence task. This identified that stimulation to only the left primary motor cortex immediately following the task significantly improved performance after 8 h or 1 day.

A further 3 papers used sotDCS, of which all stimulated bilateral F3/F4 regions and all failed to find a significant effect of sotDCS on procedural memory tasks (54, 57, 61). Both Eggert et al. (54) and Ladenbauer et al. (57) used sotDCS during a short nap after participants completed a finger tapping sequence task, and whilst neither found a significant effect of sotDCS on procedural memory, interestingly Eggert et al. found a significant decay in performance following sleep whilst Ladenbauer et al. found a significant improvement in performance following sleep. Paßmann et al. (61) similarly failed to evidence sotDCS significantly augmenting performance on a finger tapping sequence task, although did find performance increased significantly independent of condition following a full night sleep.

Rumpf et al. (77) modulated primary motor cortex activity using tACS at different frequencies immediately following a finger tapping sequence task. Performance was subsequently re-assessed 6 h after completion of the task. Interestingly, this protocol found that alpha-tACS significantly reduced performance after the 6-h interval when compared to either sham or beta-frequency tACS, suggesting a frequency specific effect of stimulation on procedural memory consolidation.

## Discussion

This systematic review aimed to explore the role of non-invasive brain stimulation in modulating different aspects of memory functioning in healthy older adults. Research was generally of a good quality, and many papers were able to generate pertinent findings. Below is a brief summary of the key findings uncovered within each memory domain, followed by a more general discussion of these findings, limitations of the current literature, and suggestions for future research.

**Figure 2:**
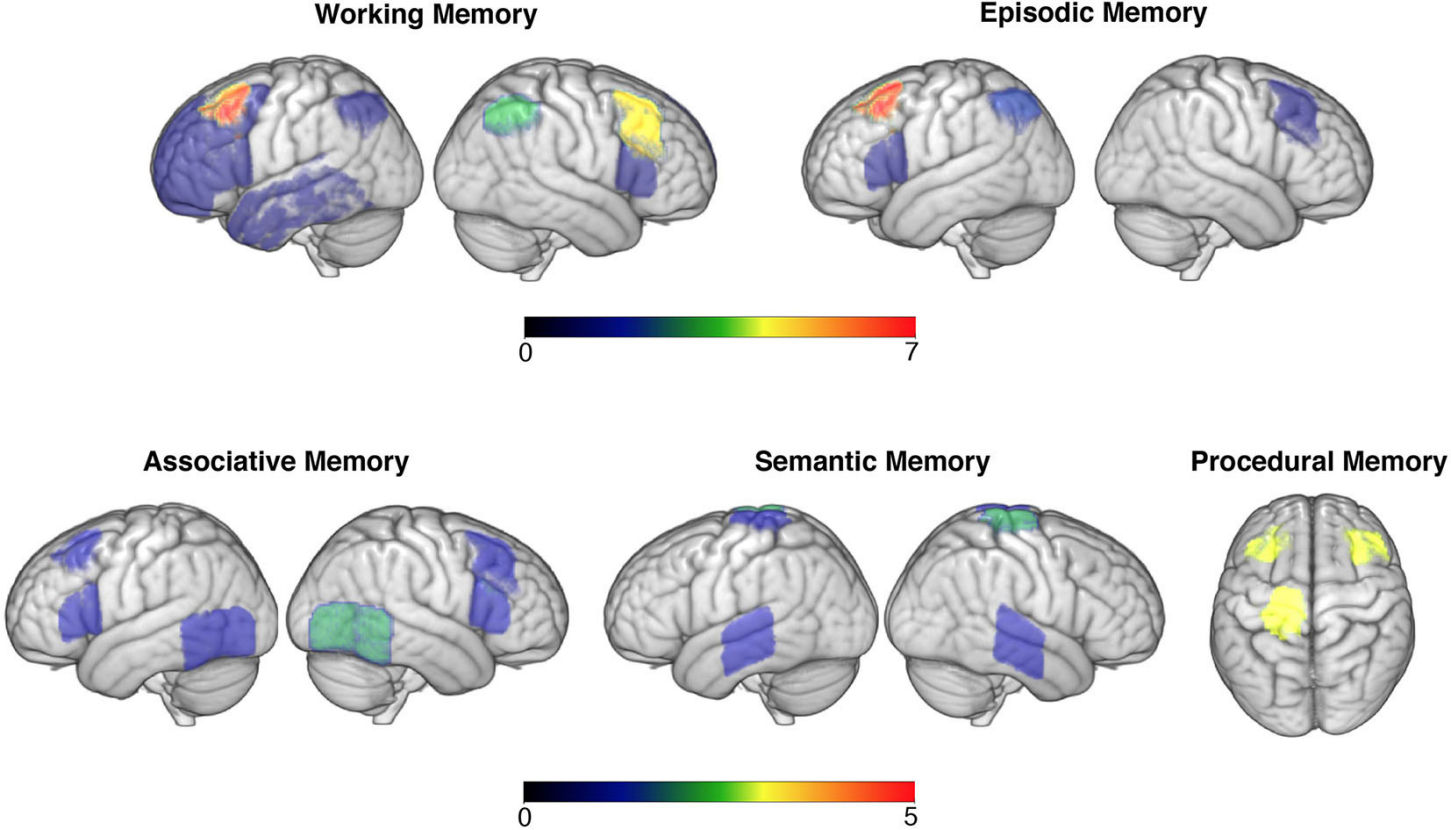
Targeted brain areas across studies are overlaid in the brain (neurological convention is used). Colour code bars indicate the number of studies that targeted a particular brain area. This figure is for illustrative purposes only.

Much non-invasive brain stimulation research focused on improving working memory competencies, with many paradigms successfully augmenting performance. Research in this domain consistently targeted prefrontal regions, with a particular preference for dorsolateral regions (presented in Figure 2). This is grounded in prior research linking this region with numerous working memory abilities during the ageing process (79). Lateral PFC regions are key areas within the frontoparietal control network (FCN), which has been shown to critically mediate working memory and attention demanding tasks (80). As such, it was unsurprising that this was a key target area along with, albeit to a lesser degree, the parietal nodes of the FCN (e.g., 50). However, not all studies showed enhanced working memory performance following DLPFC stimulation alone, with some non-significant paradigms using large samples of older adults (e.g., 29). Whether it is more efficient to target one or multiple nodes of the FCN is therefore an interesting question. The evidence from this systematic review points toward the latter, with studies targeting two regions within this network all demonstrating significant effects on working memory performance (35, 37, 39, 46), while those aiming at one specific node showing mixed results. Another pertinent question relates to the impact of stimulation delivery timing (online vs. offline). Overall, studies conducting online stimulation reported positive effects, while offline studies were less consistent.

The role of non-invasive brain stimulation on associative memory yielded more of a convoluted image. Amongst this domain, prefrontal stimulation appeared to have little, and more of an inhibitory (60), effect than with working memory tasks. More popular stimulation targets included those along the temporoparietal cortex (Figure 2), with a variety of face-name or object-location association tasks being employed. In terms of stimulation montages, those that aimed at one area were favoured in relation to those targeting several nodes within a network. Some evidence did appear to exist for stimulation of temporoparietal regions augmenting performance, which appeared to last for up to a week (e.g., 53). This is consistent with the right temporoparietal junction being particularly involved with integrating sensory and contextual stimuli (81), hence making it a likely neuroanatomical correlate for associative memory. Whilst half of the anodal tDCS or rTMS studies failed to identify this main effect, it is worth noting that of these some did identify a trend toward improvement (e.g., 55). Stimulation on sleeping participants identified less of a consistent effect, with most studies evidencing non-significant outcomes when targeting prefrontal regions; this could suggest that associative memory is less influenced by offline consolidation processes or is less amenable to improvement (61). Nevertheless, targeting frontal regions during sleep did evince some improvements in associative memory (63), providing limited support for theories that frontal regions facilitate recollection of stabilized (i.e., well-learned) memory traces (82).

Thirdly, this review explored the current state of research exploring episodic memory augmentation. Most studies uncovered a significant effect of tDCS in augmenting episodic memory, particularly in relation to word learning and recall. Most successful research in this domain targeted either the DLPFC or VLPFC (Figure 2) and appeared to identify a left hemispheric bias in processing material (e.g., 65). This appears congruent with pre-established knowledge of left prefrontal lateralisation of verbal episodic memories (83), however builds upon this by evidencing the efficacy of augmenting activity within this region in older adults. It also builds on this by adding credence to theories that the Default Mode Network (DMN) contributes toward episodic memory functioning (71), as the DMN is known to be modulated by lateral PFC sites, which are thought to support sustained attention throughout memory tasks (84). Thus, the consistent effect of tDCS in lateral PFC regions supports models of episodic memory being modulated by DMN activity and that this is susceptible to transient external modulation.

A fourth, smaller strand of research has explored whether brain stimulation could augment older adults accessing/recollecting semantic memories, primarily in the form of word generation, or landmark/celebrity naming. Interestingly all of these evidenced a significant effect, with left lateralised stimulation for verbal material proving most effective (72). This appears to be of particular relevance to older adult populations, wherein semantic recall has been shown to increasingly recruit right lateralised neural structures in later life, which is associated with worse performance on such tasks (85). Therefore, this provides evidence of older adults being less able to efficiently recruit specialized neural networks within the dominant hemisphere during memory tasks (86). Specifically, in the context of the above findings, as well as known networks of processing semantic information, it appears as though older adults were less able to recruit frontal and medial temporal lobe networks within the left hemisphere when accessing semantic memories (85). As such, external modulation of these networks could be one method of reversing this age-related trend (87). That said, it is likely that this hemispheric bias exists primarily for verbal material, as research by Ross et al. (74) found that stimulation within the right hemisphere significantly improved location recognition, suggesting a right hemispheric bias for visual learning. Interestingly, all studies focused on this memory domain opted for online stimulation protocols.

A fifth and final area assessed was the role brain stimulation may have in augmenting procedural memories. Of these studies, finger sequence tapping techniques were most commonly used, with stimulation primarily to the primary motor cortex (Figure 2). This research suggested that stimulation during or immediately after the task was most successful in augmenting performance for a period ranging from 35 min to 1 day (75, 76). It has been suggested that older adults procedural memory progressively deteriorates (88), which is corroborated by fMRI evidence of older adults being less able to effectively recruit motor cortical networks within their brain during motor tasks (89). As such, external modulation may again be one method of strengthening activity within such motor networks sub-serving motor memory. This effect may also be frequency specific, with some evidence documented here of M1 alpha-tACS specifically impeding performance (77), suggesting a significance of this frequency in M1 network connectivity and hence consolidation. Stimulation of these regions during sleep appeared to have little effect on motor performance, suggesting this network is most pertinent either during or immediately after learning motor commands (90).

Taken together, the above provides some commonalities. For example, stimulation of the DLPFC (Figure 2) appeared to provide numerous memory benefits for healthy older adults, memory research has generally been successful in identifying an effect of non-invasive brain stimulation in improving memory performance (with perhaps the exception of associative memory), and that certain neural networks (like the DMN and FCN) may constitute better targets for improving memory performance than focusing on separate nodes. Nevertheless, this review also identified some contradictory results. This comes within a context of research being of generally very good quality, using randomisation, blinding, and minimising experimenter effects. It is therefore apparent that some of the classical assumptions underlying much of this research, such as stimulation of the DLPFC alone improving memory (91), may warrant reconsideration. For instance, one way of re-interpreting this could be that the DLPFC is a vital site for attention rather than memory, which has accrued recent interest amongst brain stimulation research (92). Stimulation of this site may therefore improve one's ability to concentrate, hence this site indirectly supporting memory as information can only be encoded if attended to. This could therefore explain inconsistent findings amongst such research, as healthy individuals have been shown to vary in attentional capacity considerably later in life (93), which was not controlled for within the above studies.

Amongst the papers evaluated, numerous additional theories were posited to explain the above findings. For example, Stephens et al. (48) articulated that certain variants of the COMT genotype result in greater or diminished working memory faculties in older adults. Moreover, those with an enhanced working memory capacity COMT genotype responded preferentially to tDCS, whilst those with a diminished working memory capacity COMT genotype responded poorly to tDCS. It may therefore be plausible that variants of the COMT genotype differentially influence amenability to non-invasive brain stimulation. Such exploration of genotype-brain stimulation interactions could be extended by also exploring the role other pertinent genes play in memory. For example, the APOE gene has been recognised as potentially mediating long-term memory functioning (94), and it is interesting to note that different versions of the APOE gene result in strikingly different neural responses to brain stimulation (95). Thus, it is evident that participants may respond differently to brain stimulation as a function of gene expression and other physiological traits.

Additionally, the efficacy of brain stimulation could relate to degree of education, as Berryhill and Jones (38) identified tDCS selectively augmented working memory amongst older adults who were highly educated, whilst having little to no effect amongst those less educated. This was hypothesised to relate to better educated older adults recruiting different neural structures during working memory tasks, and would again explain individual differences in response to transcranial stimulation. These examples illustrate that both experience and genetics could impact individual's responsiveness to brain stimulation, which should be considered when assessing different outcomes observed from different samples.

A further source of inconsistency may derive from definitions of different memory modalities, both within this literature review itself as well as within primary research. Indeed, there were some discrepancies between what constituted as a test of working, associative, and episodic memory, which could result in articles being mis-represented or mis-reported. This is to be perhaps expected, given that myriad definitions of working memory alone already exist (96). Moreover, classifying memory itself can be troublesome, as memory domains can themselves be sub-divided. For example, episodic memory could be divided into verbal and visual domains which, as cited above, may each have unique neural correlates (97). Thus, it is possible that more nuanced effects exist than given credit for in this article. Nevertheless, if a semblance of order is to be given to the above research some degree of coherent memory classification will be necessary moving forward.

One alternative explanation for the above results could relate to the ageing brain functionally changing over the course of the lifespan. Increasing evidence suggests that the hemispheric biases reported earlier become less apparent as we age, with increased bilateral activity in the ageing brain (98). Brain imaging and memory research suggests that previously superior hemispheres increasingly lose their enhanced ability to process certain information over the lifespan, causing contralateral structures to increasingly activate to compensate for this loss (99). Indeed, this was explicitly highlighted within the research above. For example, older adults increasingly relied on bilateral de-differentiated neural networks during semantic memory tasks (72), instead of left hemispheric frontal-temporal networks specifically sub-serving semantic knowledge in younger individuals (85). Further evidence of more globalised activity comes from Di Rosa et al.'s (42) finding that left PFC tDCS increased bilateral PFC activity (which subsequently improved working memory performance), whilst Manenti et al. (60) showed that high performing older adults on episodic memory tasks responded equally to bilateral (rather than lateral) DLPFC stimulation. Together, this suggests bilateral hemispheric recruitment could act as a compensatory plastic strategy to support the ageing brain, in contrast to younger adult's memory faculties which might place more reliance on specialized localised circuits (100). This would also explain why paradigms such as Leach et al. (59) uncovered significant improvements in memory functioning following tDCS only with younger adults, with the above functional reorganization likely resulting in the two groups benefitting from different forms of stimulation. As such, it is possible that older adults memory networks qualitatively differ from those seen amongst younger adults.

As mentioned, there were limitations when conducting this systematic review. Firstly, due to the nature of extant literature, sample sizes included in this review were often small, which increased the risk of insufficient statistical power to yield a significant result, biased effect sizes, and unreliable results (101). This appeared to permeate the literature and may contribute toward some of the inconsistent findings observed. Furthermore, it is possible that the searching strategy missed relevant articles by its choice of terms used. Whilst this research chose to search for the term “memory” within the body of articles selected, and used similar terms to those successfully used elsewhere [e.g., (102)], it is possible that other less relevant terms, such as “learning,” could have identified a limited number of additional results. This choice equally meant papers written in other languages were excluded. Furthermore, due to the volume of included articles it has not been possible to cite all potential confounds or methodological questions within the body of this paper. Whilst quality checks were performed and summarised within this article to give an indication of the credibility of those findings reported, it is possible (although not anticipated) that bias exists within those papers cited that has not been captured here. Finally, it has been noted elsewhere that a publication bias may exist amongst studies of non-invasive brain stimulation in augmenting memory amongst those with dementia (30). This should be considered when assessing the above findings with healthy older adults, as this could distort the true landscape of brain stimulation research.

That said, the findings above do have numerous implications. Firstly, numerous research paradigms appeared to evidence a significant effect of non-invasive brain stimulation in augmenting working memory and episodic memory, both of which appear to be significantly impacted by a range of organic dementias (103). Whilst the above was conducted amongst healthy older adults, it is possible these same findings could be extrapolated to support those with early signs of pathological memory loss. Indeed, many of the same regions and mechanisms targeted by the above research are similarly implicated in Alzheimer's disease, with preliminary research showing these areas remain sensitive to non-invasive brain stimulation amongst those with dementia (104). Furthermore, whilst research included here involved “healthy” participants, many studies did not assess participants for prodromal biomarkers of dementia. It is therefore possible that many included participants may already have a predisposition to develop a dementia (105), similarly suggesting that brain stimulation may be a useful early intervention in supporting cognitive functioning. As such, an exciting future avenue for brain stimulation research may be to study if these same effects hold true amongst people with early signs of memory loss, potentially providing an opportunity to reduce the impact such changes may be having on their lives.

The above research also does much to prove that non-invasive brain stimulation is a safe, well-tolerated, and often effective approach for mitigating age-related changes in memory performance. Whilst effects varied in length, it was of particular interest to see that some paradigms were able to induce long lasting effects in memory performance. For example, Jones et al. (44) identified that 10 sessions of tDCS was sufficient to induce changes in working memory performance for up to 1-month post stimulation. Given that memory changes are something which are commonplace and distressing amongst older adults (3, 6), the potential to reduce these concerns for up to a month at a time is something which holds utility when considering the functional well-being of the ageing population. Nevertheless, it is likely that these same effects would not be possible with fewer rounds of brain stimulation, with the above research largely failing to find long-standing effects after only single doses of tDCS (e.g., 69). It is unclear as yet what exact mechanisms are associated with this dose-response curve and how dose-response will translate into risk and benefit. Nevertheless, numerous theories have been put forward to explain long-term effects, mostly related to long-term potentiation within neural networks (106). This emphasizes the need to investigate dose-response relationships when supporting maximal cognitive functioning in older adults.

This literature review also does much to identify areas where current non-invasive brain stimulation literature could expand further. Firstly, little research with healthy older adults appears to have used modalities other than tDCS, despite there being increasing evidence of the success of targeting neural networks using other approaches such as tACS (107). The application of tACS, for example, offers greater potential for individualized approaches that target networks using subject-specific tuned-frequencies [e.g., (35)]. It is also of interest to see so few articles using rTMS; this is surprising, given the extensive literature on rTMS applications to neurological and psychiatric disorders [e.g., (108, 109)]. In terms of memory domains, an area to be explored further is associative memory, as this was one domain generating particularly inconclusive findings. Whilst this does appear to be particularly susceptible to the ageing process (110, 111), many studies have failed to identify statistically significant methods of augmenting this form of memory. As such, protocols to target associative memory should be explored further to identify if it is indeed possible to reliably augment this domain. Finally, for the opposite reason, it would be of interest to explore further the impact of non-invasive brain stimulation on semantic memory, as this an area which appears to have not been explored greatly but uncovered a generally consistent effect.

To conclude, this systematic literature review has aimed to bring some degree of clarity into the current state of research into non-invasive brain stimulation in modulating healthy older adult's memory. In doing so it has uncovered that numerous approaches have been used to target five areas of memory, and that with the exception of associative memory these have uncovered a degree of consistency in effects on memory performance. Particularly established methods of augmenting memory performance included using tDCS to improve working and episodic memory performance, whilst semantic memory appeared to be similarly susceptible although less thoroughly researched. The above should be used to guide future researchers in their endeavours to better understand how to support older adults as their memory changes.

## Author Contributions

RG: conception of review, literature review, and wrote the manuscript. JR: literature review, table, and figure construction. IV: conception of review, support writing manuscript, and figure construction. All authors contributed to manuscript revisions, read, and approved the submitted version.

## Conflict of Interest

The authors declare that the research was conducted in the absence of any commercial or financial relationships that could be construed as a potential conflict of interest.
